# Monoterpenoids from the Fruits of *Amomum tsao-ko* Have Inhibitory Effects on Nitric Oxide Production

**DOI:** 10.3390/plants10020257

**Published:** 2021-01-28

**Authors:** Seong Su Hong, Ji Eun Lee, Yeon Woo Jung, Ju-Hyoung Park, Jung A. Lee, Wonsik Jeong, Eun-Kyung Ahn, Chun Whan Choi, Joa Sub Oh

**Affiliations:** 1 Bio-Center, Gyeonggido Business & Science Accelerator (GBSA), Suwon 16229, Korea; jieun@gbsa.or.kr (J.E.L.); jion123@gbsa.or.kr (Y.W.J.); lovelee90@gbsa.or.kr (J.A.L.); ws2009@gbsa.or.kr (W.J.); aek@gbsa.or.kr (E.-K.A.); cwchoi@gbsa.or.kr (C.W.C.); 2 College of Pharmacy, Dankook University, Cheonan 31116, Korea; yourselves@naver.com

**Keywords:** *Amomum tsao-ko*, monoterpenoid, anti-inflammation, nitric oxide, menthene skeleton

## Abstract

In our search for novel plant-derived inhibitors of nitric oxide (NO) with potential for treating inflammatory diseases, the phytochemicals of *Amomum tsao-ko* fruits were investigated, leading to the isolation of one bicyclic nonane (**1**), three menthene skeleton monoterpenoids (**2**–**4**), and two acyclic monoterpenoids (**5** and **6**). Their structures were identified using one- and two-dimensional nuclear magnetic resonance spectroscopy, and mass spectrometry. To the best of our knowledge, compounds **2**–**5** were obtained from the genus *Amomum* for the first time. All isolates were tested for their ability to inhibit lipopolysaccharide-stimulated NO overproduction in RAW264.7 cells. Compound **4** was found to inhibit NO production. Western blotting analysis indicated that active compound **4** can regulate inducible NO synthase expression. In addition, lipopolysaccharide-induced interleukin 1 beta and interleukin-6 overproduction was reduced in a concentration-dependent manner.

## 1. Introduction

Bioactive natural resources played a vital roles in the discovery and development of new pharmaceuticals, and many lead constituents are derived from natural products or their derivatives [1]. Plant secondary metabolites are considered an important source of bioactive natural products [2]. *Amomum* is the second largest genus in the Zingiberaceae family, comprising at least 170 species distributed across southeast Asia and northern Australia, in the Afrotropical region of Africa, the Himalayas, and the Central Pacific [3,4]. A perennial herb, *Amomum tsao-ko* Crevost and Lemarié, is used as a food additive (traditional Chinese spice) and medicine [5,6]. Its dried fruit is commonly used to treat abdominal pain, dyspepsia, malaria, nausea, throat infections, stomach disorders, vomiting, and diarrhea in the traditional medicine system [7]. Because of their numerous pharmacological activities, such as antitumor, antioxidant, and neuroprotective properties, the fruits of *A. tsao-ko* have attracted attention as a functional food and medicine [8,9,10,11,12]. The major constituents of *A. tsao-ko* are diarylheptanoid and flavonoids, which exhibit anti-oxidant, anti-tumor, anti-inflammatory and α-glucosidase inhibitory activities, neuroprotective effects, and nitric oxide (NO) inhibitory effects [13,14,15]. Inflammation is the immune system’s biological response for removing harmful stimuli and repairing damaged tissue caused through harmful factors, such as damaged cells, invasion by pathogens, irradiation or, toxic compounds [16]. When inflammation happens, the endotoxin lipopolysaccharide (LPS), produced by gram-negative bacteria, induces the expression of inducible nitric oxide synthase (iNOS), which produce excessive NO [17]. LPS also induces the release of pro-inflammatory cytokines including interleukin (IL)-1β and IL-6 which cause tissue damage and organ failure [18]; regulating these cytokines could be a therapeutic strategy for addressing various inflammatory-associated diseases. Four menthene skeleton monoterpenoids and two acyclic monoterpenoids from *A. tsao-ko* fruits have been identified as a part of an ongoing project to discover anti-inflammatory metabolites in functional plants. In LPS-induced murine macrophage RAW264.7 cells, all compounds were preliminarily screened for the ability to prevent NO production, with mechanistic studies revealing (1*R*,4*S*,6*S*)-1,6-dihydroxy-2-menthene (**4**) to be a significant anti-inflammatory constituent. In this study, we performed isolation, structural determination, and anti-inflammatory activity analysis of the isolated compounds.

## 2. Results and Discussion

### 2.1. Chemical Characterization

#### 2.1.1. Isolation Compounds

**Figure 1:**
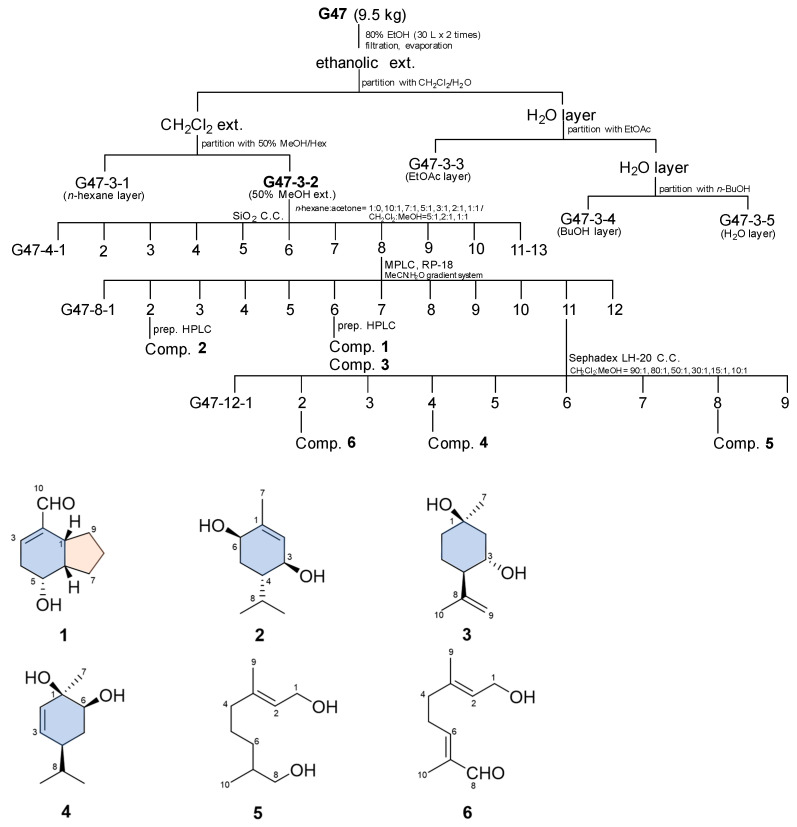
Fractionation flow map and structure of compounds isolated from the fruits of *A. tsao-ko.*

The dried fruits of *A. tsao-ko* were divided via sequential extraction with CH_2_Cl_2_ (*n*-hexane and 50% methanol (MeOH) layer), ethyl acetate, and *n*-butyl alcohol. Of the four solvent soluble fractions, the 50% MeOH fraction was performed to successive column chromatography with silica gel, medium-pressure liquid chromatography (MPLC), and preparative high-performance liquid chromatography (HPLC) to yield six compounds (Figure 1).

#### 2.1.2. Identification of Compound Structures

Compound **1** was obtained as a pale brown oil. A molecular ion peak at *m*/*z* 167 [M + H]^+^ was observed via electrospray ionization-mass spectrometry (ESI-MS) (positive ion mode). The ^1^H (Figure S2), ^13^C (Figure S3), and distortionless enhancement by polarization transfer (DEPT) nuclear magnetic resonance (NMR) spectra of **1** displayed a signal assignable to an aldehyde group [δ_H_ 9.42 (1H, s, H-10); δ_C_ 194.0] and trisubstituted olefin [δ_H_ 6.66 (s, H-3); δ_C_ 144.8 (C-2) and 146.7 (C-3)]. The ^13^C NMR and DEPT spectra indicated the presence of four methylenes (δ_C_ 32.2, 31.2, 24.9, and 24.7), two methines (δ_C_ 42.8 and 37.2), one oxymethine (δ_C_ 68.4), and an aldehyde group and double bond (Table 1). Compound **1**’s NMR data were consistent with the spectral data in the literature [19]; thus, compound **1** was identified as tsaokoin, a bicyclic nonane derivative.

As a colorless oil, compound **2** was isolated with a molecular weight of the quasi-molecular ion peak at *m*/*z* 171 [M + H]^+^, based on the ESI-MS data. The ^1^H NMR (Figure S4) data suggested H-2 as a singlet at δ_H_ 5.49; H-6, H-3, and H-4 signals appeared at δ_H_ 3.94 (brs), 3.87 (d, *J* = 9.1 Hz), and 1.61 (m), respectively; an isopropyl group at δ_H_ 2.14 (m, H-8), 1.00 (d, *J* = 7.0 Hz, Me-9), and 0.85 (d, *J* = 7.0 Hz, Me-10); and vinylic methyl signal at δ_H_ 1.79 (s, Me-7). The data revealed that compound **2** was a monoterpenoid based on the skeleton of menthene [20]. Ten signals were detected via ^13^C NMR (Figure S5) and DEPT: vinyl carbon signals C-1 and C-2 at δ_C_ 137.4 and 131.0, respectively; two oxymethines at δ_C_ 69.9 (C-3) and 68.4 (C-6); three methyls at δ_C_ 20.7 (C-7), 21.4 (C-9), and 17.0 (C-10); one methylene at δ_C_ 30.9 (C-5); and two methine signals at δ_C_ 42.8 (C-4) and 27.0 (C-8) (Table 1). The NMR results for compound **2** were compatible with the spectral data in the literature [21]; thus, compound **2** was identified as (3*S*,4*S*,6*R*)-3,6-dihydroxy-1-menthene.

Compound **3** was obtained as a pale yellowish oil. A positive ESI-MS experiment exhibited a quasi-molecular ion peak at *m*/*z* 171 [M + H]^+^. The data of ^1^H NMR (Figure S6) indicated H-9 as an *exo*-methylene singlet signals at δ_H_ 4.94 and 4.90; an oxygenated proton δ_H_ 3.84 (d, *J* = 10.5, 4.9 Hz, H-3); and two methyl signals δ_H_ 1.76 (s, CH_3_-10) and δ_H_ 1.30 (s, CH_3_-7). The ^13^C NMR (Figure S7) and DEPT spectra showed 10 signals: C-8 and C-9 *exo*-methylene carbon signals at δ_C_ 146.3 and 113.1; an oxymethine at δ_C_ 67.3 (C-3); oxygenated quaternary carbon at δ_C_ 71.4 (C-1); two methyls at δ_C_ 31.7 (C-7) and 19.3 (C-10); three methylenes at δ_C_ 46.1 (C-2), 38.1 (C-6), and 25.3 (C-5); and one methine signal at δ_C_ 54.0 (C-4) (Table 1). The NMR results for compound **3** was consistent with the spectral data in the literature [22]; thus, compound **3** were determined as (1*R*,3*S*,4*R*)-3-hydroxy isopulegol.

Compound **4** was isolated as a pale yellowish oil. The molecular weight of compound **4** was obtained via ESI-MS with *m*/*z* 171 [M + H]^+^. The ^1^H (Figure S8) and ^13^C NMR (Figure S9) spectroscopic data were similar to those of compound **2**, indicating that they have similar structures. The ^1^H NMR data indicated H-2 and H-3 as a pair olefinic double bond with signals at δ_H_ 5.71 (2H, m); oxygenated proton δ_H_ 3.49 (brd, *J* = 12.6 Hz, H-6); isopropyl group at δ_H_ 1.70 (m, H-8), 0.94 (d, *J* = 7.0 Hz, Me-9), and 0.91 (d, *J* = 7.0 Hz, Me-10); and methyl group at δ_H_ 1.36 (s, Me-7). The ^13^C NMR and DEPT spectra showed 10 signals: C-3 and C-2 olefinic double bond signals at δ_C_ 134.1 and 132.1, respectively; oxymethine at δ_C_ 73.6 (C-6); oxygenated quaternary carbon at δ_C_ 69.1 (C-1); three methyls at δ_C_ 25.9 (C-7), 19.6 (C-9), and 19.0 (C-10); one methylene at δ_C_ 30.1 (C-5); and two methine signals at δ_C_ 42.6 (C-4) and 31.5 (C-8) (Table 1). The NMR data of compound **4** were consistent with the spectral data in the literature [23]; thus, compound **4** was determined as (1*R*,4*S*,6*S*)-1,6-dihydroxy-2-menthene.

Compound **5** was obtained as a colorless oil. The molecular weight of compound **5** was observed via ESI-MS with *m/z* 173 [M + H]^+^. The ^1^H NMR (Figure S10) data indicated H-2 as a double bond at δ_H_ 5.43 (t, *J* = 6.3 Hz); two oxymethylene protons at δ_H_ 4.17 (d, *J* = 6.3 Hz, H_2_-1), 3.52 (d, *J* = 10.5, 6.3 Hz, H_a_-8), and 3.46 (d, *J* = 9.8, 6.3 Hz, H_b_-8); olefinic methyl group at δ_H_ 1.69 (s, CH_3_-9); and tertiary methyl group at δ_H_ 0.94 (d, *J* = 7.0 Hz, CH_3_-10). The ^13^C NMR (Figure S11) and DEPT spectra showed 10 signals: C-3 and C-2 olefinic carbon signals at δ_C_ 139.9 and 123.4, respectively; two oxymethylenes at δ_C_ 68.3 (C-8) and 59.4 (C-1); two methyl signals at δ_C_ 16.5 (C-10) and 16.2 (C-9); three methylene carbons at δ_C_ 39.7 (C-4), 32.6 (C-6), and 24.9 (C-5); and one methine signal at δ_C_ 35.6 (C-7) (Table 1). The NMR spectral data of compound **5** agreed with those reported in the literature [24]; thus, compound **5** was identified as 3,7-dimethyl-2-octene-1,8-diol.

Compound **6** was isolated as a colorless oil with a molecular weight of a quasi-molecular ion peak at *m*/*z* 169 [M + H]^+^, based on the ESI-MS data. The ^1^H NMR (Figure S12) data indicated H-2 and H-6 as a double bond at δ_H_ 5.39 (t, *J* = 7.0 Hz) and 6.40 (t, *J* = 7.0 Hz), respectively; aldehyde singlet at δ_H_ 9.32 (s, H-8); oxymethylene signal at δ_H_ 4.12 (d, *J* = 7.0 Hz, H_2_-1); and two vinylic methyl groups at δ_H_ 1.68 (s, CH_3_-10) and 1.63 (s, CH_3_-9). The ^13^C NMR (Figure S13) and DEPT spectra showed 10 signals: C-6, C-7, C-3, and C-2 vinylic carbon signals at δ_C_ 153.7, 139.6, 137.9 and 124.5, respectively; aldehyde group at δ_C_ 195.3 (C-8); oxymethylene at δ_C_ 59.3 (C-1); two methyl groups at δ_C_ 16.3 (C-9) and 9.3 (C-10); and two methylenes at δ_C_ 37.8 (C-4) and 27.1 (C-5) (Table 1). The MS and NMR data of compound **6** agreed well with those reported in the literature [25]; thus, compound **6** was identified as 8-oxogeraniol.

### 2.2. Nitric Oxide Inhibition Activity and Cell Viability

To obtain plant-derived inhibitors of NO as potential lead compounds for treating inflammation disorders, monoterpene constituents **1**–**6** isolated from the fruits of *A. tsao-ko* were assayed to determine their inhibitory activities on LPS-induced NO production in murine macrophage RAW 264.7 cells via the Griess reaction assay, as described previously [26]. The IC_50_ value of NG-methyl-L-arginine acetate used as a positive control was 34.2 μM. All isolates were tested for their inhibitory effects on LPS-induced NO generation and the IC_50_ values were included in Table 2. Based on their IC_50_ values, compound **4** moderately inhibited toward LPS-mediated NO overproduction, with an IC_50_ values of 82.5 μM, whereas the other constituents showed essentially no efficacy (Table 2 and Figure S1). Cell viability was evaluated via the (3-(4,5-dimethylthiazol-2-yl)-2,5-diphenyltetrazolium bromide (MTT) assay. All compounds exhibited no cytotoxicity at effective concentration for inhibiting NO production in LPS-stimulated macrophage cells (Table 2).

### 2.3. Evaluation of iNOS Protein Expression and Pro-Inflammatory Cytokine mRNA Expressions

**Figure 2:**
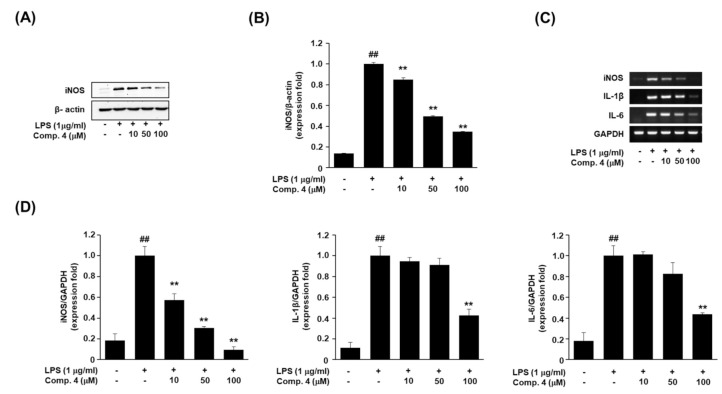
Effects of (1*R*,4*S*,6*S*)-1,6-dihydroxy-2-menthene (compound **4**) on iNOS protein and pro-inflammatory cytokine mRNA expression in LPS-stimulated RAW264.7 cells. RAW264.7 cells were pretreated with different concentrations (10, 50, 100 μM) of compound **4** for 1 h, and then stimulated with 1 µg/mL LPS for 24 h. (**A**,**B**) Western blot analysis of iNOS expression in cells treated with compound 4 (10, 50, 100 μM). β-Actin was used as a loading control. (**C**,**D**) Levels of iNOS, IL-1β, and IL-6 mRNAs were determined via RT-PCR. RT-PCR data results were expressed as the fold of changes of the target gene (iNOS, IL-1β, and IL-6) and normalized to the GAPDH. Values represent the mean ± SD of three independent experiments. Statistical significance is indicated (^##^ *p* < 0.01 compared to the untreated control/LPS (-), while ** *p* < 0.01, compared to LPS-treated cells group/LPS (+)).

L-Arginine-derived NO is an intracellular signaling molecule formed in mammalian cells by different three isoforms of nitric oxide synthase (NOS). The isozymes are referred to as neuronal NOS (nNOS or NOS I), inducible NOS (iNOS or NOS II), and an endothelial NOS (eNOS or NOS III) [27]. Excessive generation of NO by iNOS is seen in inflammatory diseases such as autoimmune and chronic inflammatory disorders [28]. To examine the mechanism of NO inhibition by active compound **4**, we evaluated iNOS protein expression via western blotting. As shown in Figure 2A,B, treatment of RAW264.7 cells with LPS (1 μg/mL) remarkably increased iNOS expression. However, pretreatment with compound **4** significantly and dose-dependently inhibited iNOS expression.

Next, we confirmed the effect of compound **4** on the expression of inflammatory factors, i.e., iNOS, IL-1β, and IL-6 in LPS-stimulated RAW264.7 cells. IL-1β and IL-6 are the most important pro-inflammatory cytokines in an inflammatory response. The inhibition of pro-inflammatory cytokine, such as IL-1β and IL-6 is essential for the control of an inflammatory response [29]. We measured the levels of the relevant mRNAs via reverse transcription polymerase chain reaction (RT-PCR) [30]. Like the iNOS protein expression result, compound **4** treatment decreased the iNOS expression at the mRNA level, in a dose-dependent manner. And reduced IL-1β and IL-6 mRNA level (Figure 2C,D). These results of the experiment with RT-PCR need to be verified through Real-time PCR in order to measure the expression level more correctly in the further study [31].

*A. tsao-ko* is an active, traditional herb medicine used to treat various inflammatory diseases [32]. The present study was undertaken to elucidate the pharmacological active molecule from the fruits of *A. tsao-ko* on the production of inflammatory mediators in macrophages. We showed that (1*R*,4*S*,6*S*)-1,6-dihydroxy-2-menthene (4) isolated from *A. tsao-ko* suppressed the production of NO, iNOS, IL-1β, and IL-6 in LPS-stimulated RAW264.7 cells, which are primary peritoneal macrophages. This suppression correlated with downregulated gene expression of IL-1β, IL-6, and, iNOS. NO, which are produced by iNOS, have been implicated as important mediators in endotoxemia and inflammatory conditions. However, although the anti-inflammatory effects of (1*R*,4*S*,6*S*)-1,6-dihydroxy-2-menthene (4) were identified, their exact mechanism of action was not determined. Thus, the active constituent can be further studied for their possible inhibitory mechanism toward the proinflammatory cytokines as well as they can be tested in the in vivo inflammatory models.

## 3. Materials and Methods

### 3.1. General

The optical rotations were measured with a JASCO P-2000 polarimeter (Tokyo, Japan). 1D and 2D NMR experiments were conducted on a Bruker Ascend III 700 instrument (Bruker-Biospin, Karlsruhe, Germany) with tetramethylsilane as an internal reference; chemical shifts are expressed in δ values. ESI masses were acquired on a LTQ Orbitrap XL mass spectrometer (Thermo Fisher Scientific, Waltham, MA, USA). MPLC (Teledyne ISCO, Lincoln, NE, USA) separations were carried out on a RediSep^®^ Rf column (Teledyne ISCO, Lincoln, NE, USA). Preparative HPLC was conducted on an LC-8A chromatography system (two LC-8A solvent delivery unit, an SPD-M20A photodiode array detector, and a CBM-20A communication module; Shimadzu, Kyoto, Japan) and J’sphere ODS-H80 column (4 μm, 250 × 20 mm, YMC Corp., Kyoto, Japan). The SPECTRAmax system was used as an ELISA reader (Molecular Devices, Sunnyvale, CA, USA). Thin layer chromatography (TLC) was carried out using ALUGRAM^®^ SIL G/UV_254+366_ (0.2 mm, Macherey-Nagel GmbH & Co. KG, Düren, Germany) plates, and spots were visualized with 10% vanillin−sulfuric acid reagent. All other chemicals, reagents, and solvents were of analytical grades.

### 3.2. Source of Plant Material

The fruits of *A. tsao-ko* were purchased in February 2012 from the Seoul Yangnyeong Market (Seoul, Korea) and authenticated by the corresponding authors (Prof., J.S.O.). A voucher specimen (G47) has been deposited in the Herbarium at the College of Pharmacy, Dankook University, Korea.

### 3.3. Extraction and Isolation of Compounds

The dried fruits of *A. tsao-ko* (5.0 kg) were extracted twice with 80% ethanol (36 L) at room temperature as around 21–25 °C for 2 days, which yielded the ethanolic extract (219 g). The ethanolic extract was then suspended in H_2_O and partitioned successively with CH_2_Cl_2_ (2 × 5 L), ethyl acetate (2 × 5 L), and *n*-butyl alcohol (2 × 5 L). The CH_2_Cl_2_-soluble fraction was suspended in *n*-hexane and partitioned with solvent to obtain 50% MeOH (2 × 5 L). The 50% MeOH fraction showed inhibitory activity with an IC_50_ below 25 μg/mL on NO overproduction, and thus was subjected to further isolation. The 50% MeOH fraction (3.5 g) was chromatographed on a silica gel column, using a step-wise gradient solvent system of *n*-hexane−acetone (1:0 to 1:1, *v*/*v*) and CH_2_Cl_2_−MeOH (5:1 to 1:1, *v*/*v*), to yield 13 fractions (G47–4–1 to G47–4–13) according to TLC analysis. Fraction G47–4–8 (1.0 g) was fractionated using MPLC (RediSep^®^ Rf silica gel 100 gram, 75 mL/min, *n*-hexane−CHCl_3_; 90:10–70:30, *v*/*v*, 100 min) to afford subfraction G47–8–1 to G47–8–12. Compound **2** (17.5 mg) was isolated from the above subfraction, G47–8–2, via preparative HPLC (MeCN−H_2_O, 75:25 to 100:0, *v*/*v*, 12 mL/min, 40 min). Subsequent purification of G47–8–6 with the same HPLC conditions (MeCN−H_2_O, 70:30 to 100:0, *v*/*v*, 12 mL/min, 40 min) resulted in compounds **1** (38.6 mg) and **3** (18.1 mg). Fraction G47–8–11 (0.8 g) was subjected to further chromatographic separation on a Sephadex LH-20 column and eluted with a step-wise gradient of CH_2_Cl_2_−MeOH (80:1 to 10:1, *v*/*v*), to yield compounds **4** (7.8 mg), **5** (3.1 mg), and **6** (4.5 mg) based on TLC. The isolation flow map and chemical structures of the six monoterpenoids isolated from the fruits of *A. tsao-ko* are shown in Figure 1.

#### 3.3.1. Tsaokoin (**1**)

Pale brown oil; ${\left[\alpha \right]}_{D}^{25}$ ‒1.7° (c 0.3, CH_2_Cl_2_); UV (CH_3_OH) λ_max_ (log ε) 233 (4.1) nm; ^1^H NMR (700 MHz, CDCl_3_) δ 9.42 (1H, s, H-10), 6.66 (1H, brs, H-3), 4.06 (1H, t-like, *J* = 4.9 Hz, H-5), 2.99 (1H, d, *J* = 7.0 Hz, H-1), 2.54 (1H, td, *J* = 18.2, 4.9 Hz, H-4), 2.44 (1H, m, H-6), 2.12 (1H, m, H_a_-9), 1.80 (2H, m, H-8), 1.57 (2H, m, H-7), 1.43 (1H, m, H_b_-9); ^13^C NMR data, see Table 1; ESI-MS (positive ion mode): *m*/*z* 167 [M + H]^+^.

#### 3.3.2. (3*S*,4*S*,6*R*)-3,6-Dihydroxy-1-Menthene (**2**)

Colorless oil; ${\left[\alpha \right]}_{D}^{25}$ +9.3° (c 0.4, CH_3_OH); ^1^H NMR (700 MHz, methanol-*d_4_*) δ 5.49 (1H, s, H-2), 3.94 (1H, brs, H-6), 3.87 (1H, d, *J* = 9.1 Hz, H-3), 2.14 (1H, m, H-8), 1.79 (3H, s, H-7), 1.74 (1H, dt, *J* = 14.0, 2.8 Hz, H_a_-5), 1.61 (1H, m, H-4), 1.41 (1H, td, *J* = 13.3, 4.2 Hz, H_b_-5), 1.00 (3H, d, *J* = 7.0 Hz, H-9), 0.85 (3H, d, *J* = 7.0 Hz, H-10); ^13^C NMR data, see Table 1; ESI-MS (positive ion mode): *m*/*z* 171 [M + H]^+^.

#### 3.3.3. (1*R*,3*S*,4*R*)-3-Hydroxy Isopulegol (**3**)

Pale yellowish oil; ${\left[\alpha \right]}_{D}^{25}$ +15.5° (c 0.5, CH_2_Cl_2_); ^1^H NMR (700 MHz, CDCl_3_) δ 4.94 (1H, s, H_a_-9), 4.90 (1H, s, H_b_-9), 3.84 (1H, td, *J* = 10.5, 4.9 Hz, H-3), 2.09 (1H, ddd, *J* = 13.3, 4.2, 2.8 Hz, H_a_-2), 1.91 (1H, m, H-4), 1.76 (3H, s, H-10), 1.71 (2H, m, H_a_-5), 1.66 (1H, ddd, *J* = 14.0, 6.3, 2.8 Hz, H_a_-6), 1.57 (1H, ddd, *J* = 13.3, 7.0, 4.2 Hz, H_b_-5), 1.43 (1H, td, *J* = 14.0, 4.2 Hz, H_b_-6), 1.36 (1H, dd, *J* = 12.6, 11.2 Hz, H_b_-2), 1.30 (3H, s, H-7); ^13^C NMR data, see Table 1; ESI-MS (positive ion mode): *m/z* 171 [M + H]^+^.

#### 3.3.4. (1*R*,4*S*,6*S*)-1,6-Dihydroxy-2-Menthene (**4**)

Pale yellowish oil; ${\left[\alpha \right]}_{D}^{25}$ +5.1° (c 0.1, CH_2_Cl_2_); ^1^H NMR (700 MHz, CDCl_3_) δ 5.71 (2H, overlap, H-2, 3), 3.49 (1H, brd, *J* = 12.6 Hz, H-6), 2.10 (1H, m H-4), 1.81 (1H, m H_b_-5), 1.70 (1H, m H-8), 1.40 (1H, m H_b_-5), 1.36 (3H, s H-7), 0.94 (3H, d, *J* = 7.0 Hz, H-9), 0.91 (3H, d, *J* = 7.0 Hz, H-10); ^13^C NMR data, see Table 1; ESI-MS (positive ion mode): *m/z* 171 [M + H]^+^.

#### 3.3.5. 3,7-Dimethyl-2-Octene-1,8-Diol (**5**)

Colorless oil; ^1^H NMR (700 MHz, CDCl_3_) δ 5.43 (1H, t, *J* = 6.7 Hz, H-2), 4.17 (2H, d, *J* = 6.3 Hz, H-1), 3.52 (1H, dd, *J* = 10.5, 6.3 Hz, H_a_-8), 3.46 (1H, dd, *J* = 9.8, 6.3 Hz, H_b_-8), 2.05 (2H, m, H-4), 1.69 (3H, s, H-9), 1.66 (1H, s, H-7), 1.52 (1H, m, H_a_-5), 1.42 (1H, m H_a_-6), 1.38 (1H, m H_b_-5), 1.11 (1H, s H_b_-6), 0.94 (3H, d, *J* = 7.0 Hz, H-10); ^13^C NMR data, see Table 1; ESI-MS (positive ion mode): *m/z* 173 [M + H]^+^.

#### 3.3.6. 8-Oxogeraniol (**6**)

Colorless oil; ^1^H NMR (700 MHz, CDCl_3_) δ 9.32 (1H, s, H-8), 6.40 (1H, t, *J* = 7.0 Hz, H-6), 5.39 (1H, t, *J* = 7.0 Hz, H-2), 4.12 (2H, d, *J* = 7.0 Hz, H-1), 2.43 (2H, dd, *J* = 15.4, 7.7 Hz, H-5), 2.16 (2H, t, *J* = 7.7 Hz, H-4), 1.68 (3H, s, H-10), 1.63 (3H, s, H-9); ^13^C NMR data, see Table 1; ESI-MS (positive ion mode): *m/z* 169 [M + H]^+^.

### 3.4. Anti-Inflammatory Activities

#### 3.4.1. Cell Culture Conditions

Mouse RAW264.7 macrophage cells (TIB-71) were maintained in Dulbecco’s modified Eagle medium (DMEM) supplemented with 10% fetal bovine serum (FBS), 100 U/mL penicillin, and 0.1 mg/mL streptomycin in a humidified incubator with 5% CO_2_ at 37 °C.

#### 3.4.2. Measurement of LPS-Induced NO Production and MTT Assay for Cell Viability

The Griess reaction was performed to measure the concentration of nitrite in the medium as an indicator of NO production. RAW264.7 macrophage cells were cultured in a 96-well plate after seeding at a density of 4 × 10^5^ cells/well for 24 h in DMEM supplemented with 10% FBS, and stimulated with or without LPS (1 μg/mL, Sigma Aldrich, St. Louis, MO, USA) in the presence or absence of the compounds. After 24 h of incubation at 37 °C, 5% CO_2_, the cell supernatant was reacted with equal volumes of Griess reagent solutions to determine nitrite production. Absorbance was measured with a microplate reader (Molecular Devices, Sanjose, CA, USA) at 540 nm. Cell viability was confirmed via MTT (Duchefa Biochemie, Haarlem, The Netherlands) assay. The supernatant was removed and medium containing MTT solution (5 mg/mL in phosphate-buffered saline) was added to each well and incubated for 2 h. The medium was removed, and 100 μL of dimethyl sulfoxide (Duchefa Biochemie, Haarlem, The Netherlands) was added to each well to dissolve the purple formazan product to obtain a colored solution. Absorbance was measured at 540 nm with a microplate reader.

#### 3.4.3. Reverse Transcription-Polymerase Chain Reaction (RT-PCR)

RAW264.7 cells, plated in 6-well plates (1 × 10^6^ cells/well), were treated with compound **4** (10, 50, and 100 μM) for 1 h prior to LPS, and stimulated with 1 μg/mL LPS or remained unstimulated for 24 h. Total RNA was isolated using TRIzol^®^ reagent (Invitrogen, Carlsbad, CA, USA). RNA (1 μg) was used as a template for each reverse-transcribed using a SuperScript^®^III First-Strand Synthesis System (Invitrogen, Carlsbad, CA, USA). Polymerase chain reaction was performed at 95 °C for 5 min (1 cycle); 95 °C for 30 s, 55 °C for 40 s and 72 °C for 1 min (30 cycles); and final extension at 72 °C for 10 min. The primers for PCR were synthesized by Bioneer Corporation (Daejeon, Korea). Glyceraldehyde 3-phosphate dehydrogenase (GAPDH, housekeeping gene) was used as an internal reference control. The PCR primer sequences are shown in Table 3. The bands of interest were quantified using the ChemiDoc XRS system and Quantity One software (Bio-Rad Laboratories, Hercules, CA, USA).

#### 3.4.4. Western Blot Analysis

RAW264.7 cells, plated in 6-well plates (1 × 10^6^ cells/well), were treated with compound **4** (10, 50, and 100 μM). Next, the cells were resuspended and lysed in RIPA buffer (Sigma Aldrich, St. Louis, MO, USA) including protease inhibitors (Sigma Aldrich, St. Louis, MO, USA). The cell lysates were clarified via centrifugation at 15,000× *g* for 30 min at 4 °C, and the lysates were subjected to western blot analysis as previously described [33]. Protein expression was analyzed via immunoblotting with antibodies against anti-iNOS (cat. no. ab3523; dilution, 1:500, Abcam, Cambridge, UK) and β-actin (cat. no. 5125; dilution, 1:1000, Cell Signaling Technology, Danvers, MA, USA). All western blot results are representative of at least three independent experiments. The bands of interest were quantified using the imageJ software.

#### 3.4.5. Statistical Analysis

Data are expressed as mean ± standard deviation (SD). The results were analyzed for statistical significance using Student’s *t*-test and one-way analysis of variance. Values of * *p* < 0.05, ** *p* < 0.01 were considered statistically significant.

## 4. Conclusions

In this study, we performed phytochemical and biological activity analysis of monoterpene constituents isolated from *A. tsao-ko* fruits. The isolated compounds constituted one bicyclic nonane (**1**), three menthene skeleton monoterpenoids (**2**–**4**), and two acyclic monoterpenoids (**5** and **6**). To the best of our knowledge, compounds **2**–**5** were obtained from the genus *Amomum* for the first time. Among these compounds, compound **4** ((1*R*,4*S*,6*S*)-1,6-dihydroxy-2-menthene) exerts the anti-inflammatory effect by inhibiting the NO production via down-regulation of iNOS in LPS-stimulated RAW264.7 cells. This study is the first attempted to reveal the anti-inflammatory effect of compound **4** isolated from *A. tsao-ko*, we also can provide evidence that compound **4** suppressed the expression of pro-inflammatory cytokines, such as IL-1β and IL-6 in LPS-induced macrophages. However, further studies using anti-inflammatory drugs are required to estimate the efficiency of compound **4** on its anti-inflammatory potential. The discovery of these functional monoterpenoids suggests that the fruits of *A. tsao-ko* have medicinal value for treating inflammation and related disorders.

## Figures and Tables

**Table 1:** ^13^C NMR spectroscopic data of **1**–**6** (δ in ppm, 175 MHz, in CDCl_3_) *^a^*.

Position	1	2 *^b^*	3	4	5	6
1	37.2 d *^c^*	137.4 s	71.4 s	69.1 s	59.4 t	59.3 t
2	144.8 s	131.0 d	46.1 t	132.1 d	123.4 d	124.5 d
3	146.7 d	69.9 d	67.3 d	134.1 d	139.9 s	137.9 s
4	31.2 t	42.8 d	54.0 d	42.6 d	39.7 t	37.8 t
5	68.4 d	30.9 t	25.3 t	30.1 t	24.9 t	27.1 t
6	42.8 d	68.4 d	38.1 t	73.6 d	32.6 t	153.7 d
7	24.7 t	20.7 q	31.7 q	25.9 q	35.6 d	139.6 s
8	24.9 t	27.0 d	146.3 s	31.5 d	68.3 t	195.3 d
9	32.2 t	21.4 q	113.1 t	19.6 q	16.2 q	16.3 q
10	194.0 d	17.0 q	19.3 q	19.0 q	16.5 q	9.3 q

*^a^* Assignments were confirmed by 1D and 2D NMR data. *^b^* Data were measured in methanol-*d*_4_. *^c^* Carbon multiplicity deduced by DEPT and HSQC.

**Table 2:** Inhibitory effects of isolated compounds on LPS-induced NO production in RAW 264.7 cells.

Compound	IC_50_ (μM) *^a^*	MTT (μM)
tsaokoin (**1**)	>100	>100
(3*S*,4*S*,6*R*)-3,6-dihydroxy-1-menthene (**2**)	>100	>100
(1*R*,3*S*,4*R*)-3-hydroxy isopulegol (**3**)	>100	>100
(1*R*,4*S*,6*S*)-1,6-dihydroxy-2-menthene (**4**)	82.5	>100
3,7-dimethyl-2-octene-1,8-diol (**5**)	>100	>100
8-oxogeraniol (**6**)	>100	>100
NG-methyl-L-arginine acetate *^b^*	34.2	>100

*^a^* IC_50_ represents the concentration of an inhibitor required for half maximal inhibition. *^b^* NG-methyl-L-arginine acetate was used as a positive control.

**Table 3:** Primer sequence used to detect cytokine gene expression.

Gene	Primer Sequences	
iNOS	forward reverse	5′-GAGTTCGAGACTTCTGTGA-3′ 5′-GGCGATCTGGTAGTAGTAG-3′
IL-1β	forward reverse	5′-CTTTGAAGAAGAGCCCATCC-3′ 5′ TTTGTCGTTGCTTGGTTCTC 3′
IL-6	forward reverse	5′-CACTTCACAAGTCGGAGGCTT-3′ 5′-GCAAGTGCATCATCGTTGTTC-3′
GAPDH	forward reverse	5′-CAGGTAAACTCAGGAGAGTG-3′ 5′-GTAGACTCCACGACATACTC-3′

## Supplementary Materials

The following are available online at https://www.mdpi.com/2223-7747/10/2/257/s1, Figure S1: Effect of **4** on cell viability and NO production, Figure S2: ^1^H NMR spectrum of **1**, Figure S3: ^13^C NMR spectrum of **1**, Figure S4: ^1^H NMR spectrum of **2**, Figure S5: ^13^C NMR spectrum of **2**, Figure S6: ^1^H NMR spectrum of **3**, Figure S7: ^13^C NMR spectrum of **3**, Figure S8: ^1^H NMR spectrum of **4**, Figure S9: ^13^C NMR spectrum of **4**, Figure S10: ^1^H NMR spectrum of **5**, Figure S11: ^13^C NMR spectrum of **5**, Figure S12: ^1^H NMR spectrum of **6**, Figure S13: ^13^C NMR spectrum of **6**.
