# Supplementary material for: Monoterpenoids from the Fruits of Amomum tsao-ko Have Inhibitory Effects on Nitric Oxide Production
Source: Plants (Basel). 2021 Jan 28;10(2):257. doi: 10.3390/plants10020257 (PMC7911220; doi:10.3390/plants10020257)

Monoterpenoids from the Fruits of *Amomum tsao-ko* Have Inhibitory Effects on Nitric Oxide Production

Seong Su Hong [1,*], Ji Eun Lee [1], Yeon Woo Jung [1], Ju-Hyoung Park [2], Jung A. Lee [1], Wonsik Jeong [1], Eun-Kyung Ahn [1], Chun Whan Choi [1] and Joa Sub Oh [2,*]

[1] *Bio-Center, Gyeonggido Business & Science Accelerator (GBSA), Suwon 16229, Korea.*

[2] *College of Pharmacy, Dankook University, Cheonan 31116, Korea.*

.

SUPPORTING INFORMATION

List of Supporting Information **Page**

Figure S1. Effect of (1*R*,4*S*,6*S*)-1,6-dihydroxy-2-menthene (compound **4**) on cell viability and NO production in LPS-stimulated RAW264.7 cells. …....................................…………S3

Figure S2. The ^{1}H NMR spectrum of **1** …...………… S4

Figure S3. The ^{13}C NMR spectrum of **1**..…..........…....... S4

Figure S4. The ^{1}H NMR spectrum of **2** …...………… S5

Figure S5. The ^{13}C NMR spectrum of **2**…..…....... S5

Figure S6. The ^{1}H NMR spectrum of **3** …...………… S6

Figure S7. The ^{13}C NMR spectrum of **3**…..…....... S6

Figure S8. The ^{1}H NMR spectrum of **4** …..……… S7

Figure S9. The ^{13}C NMR spectrum of **4**…..…....... S7

Figure S10. The ^{1}H NMR spectrum of **5** …...……S8

Figure S11. The ^{13}C NMR spectrum of **5**…..…....... S8

Figure S12. The ^{1}H NMR spectrum of **6**.......................................…...........……… S9

Figure S13. The ^{13}C NMR spectrum of **6**..…..............…....... S0

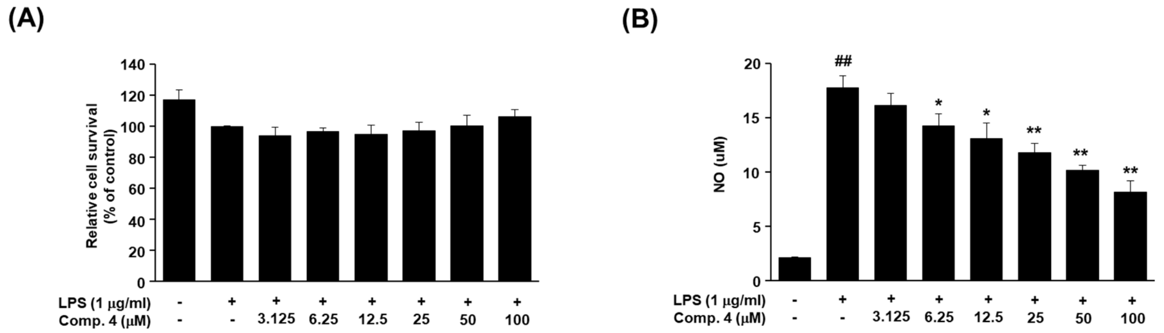

Figure S1. Effect of (1*R*,4*S*,6*S*)-1,6-dihydroxy-2-menthene (compound 4) on cell viability and NO production in LPS-stimulated RAW264.7 cells. The cells were pre-treated with compound 4 (3.125–100 μM) for 1 h and stimulated with LPS (1 μg/mL) for 24 h. (A) Cell viability, as determined using the MTT assay. (B) NO production in the cell culture supernatant, as measured using the Griess reagent. Values represent the mean ± SD of three independent experiments. Statistical significance is indicated ($^{\#\#}p < 0.01$ compared to the untreated control/LPS (−), while $^{*}p < 0.05$, $^{**}p < 0.01$, compared to LPS-treated cells group/LPS (+)).

Figure S2.

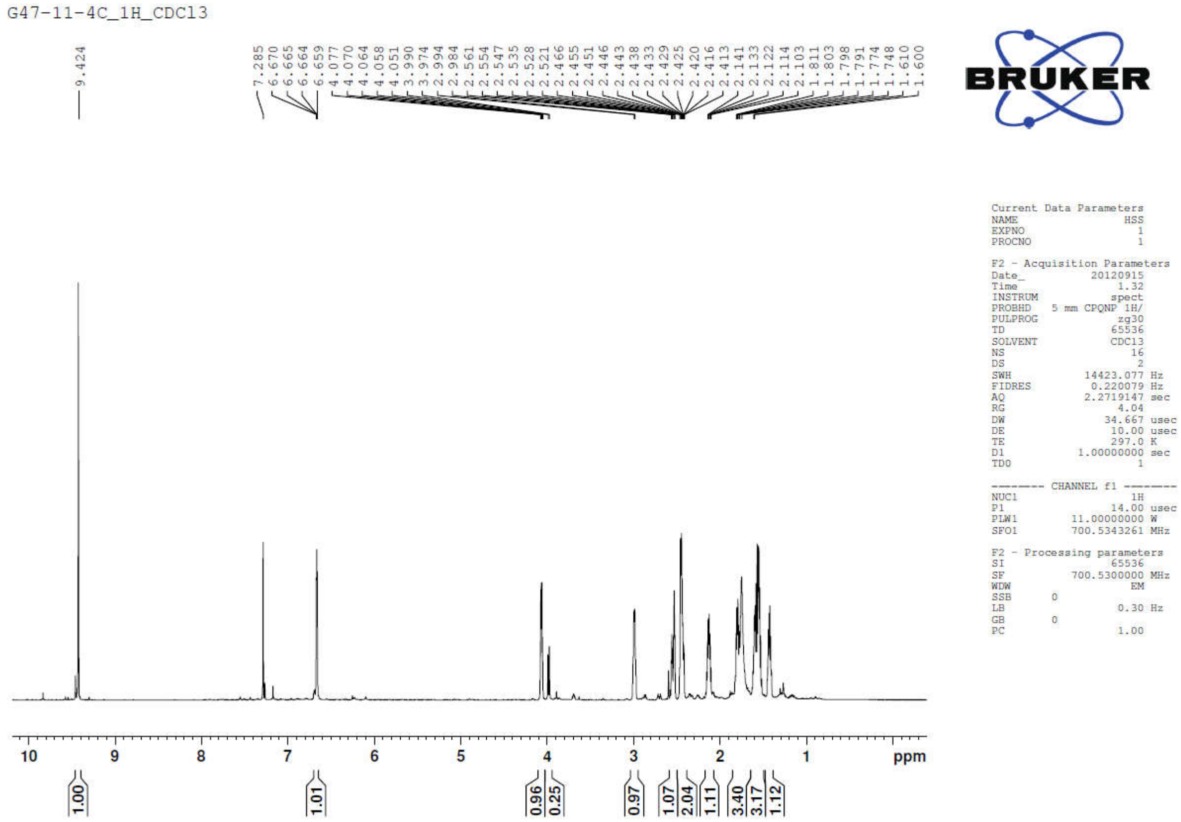

Figure S3.

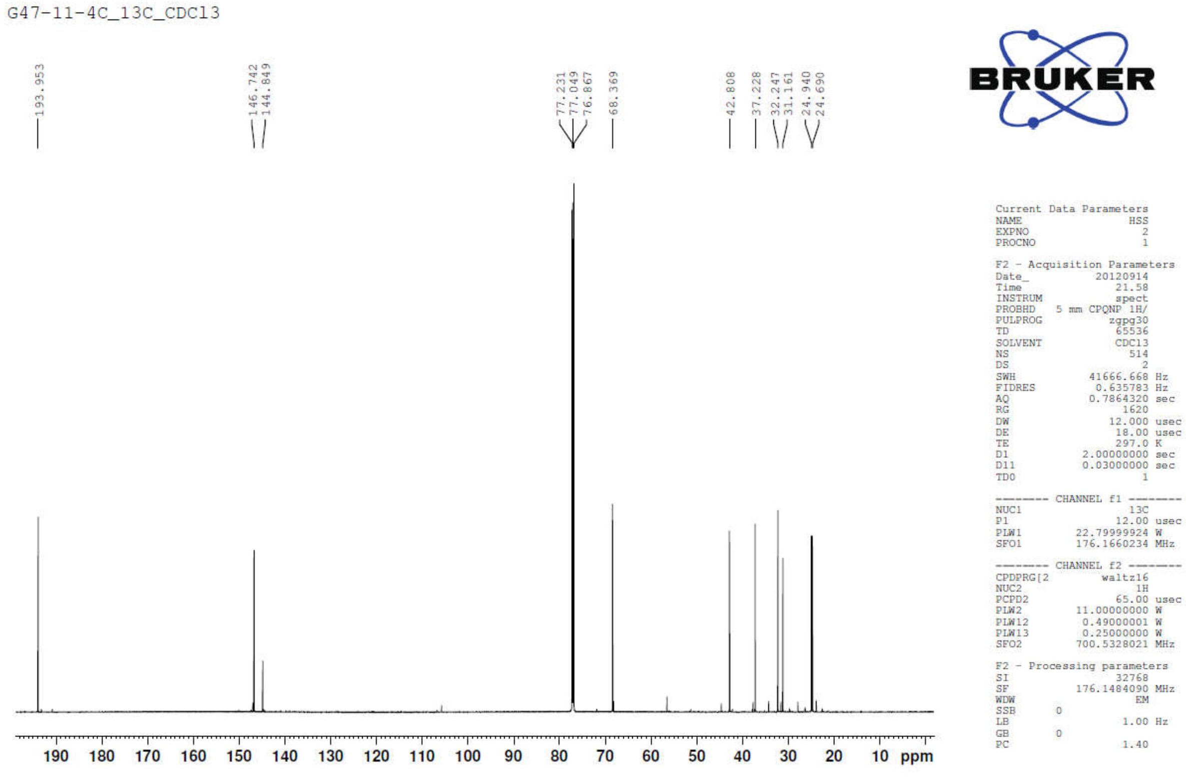

Figure S4.

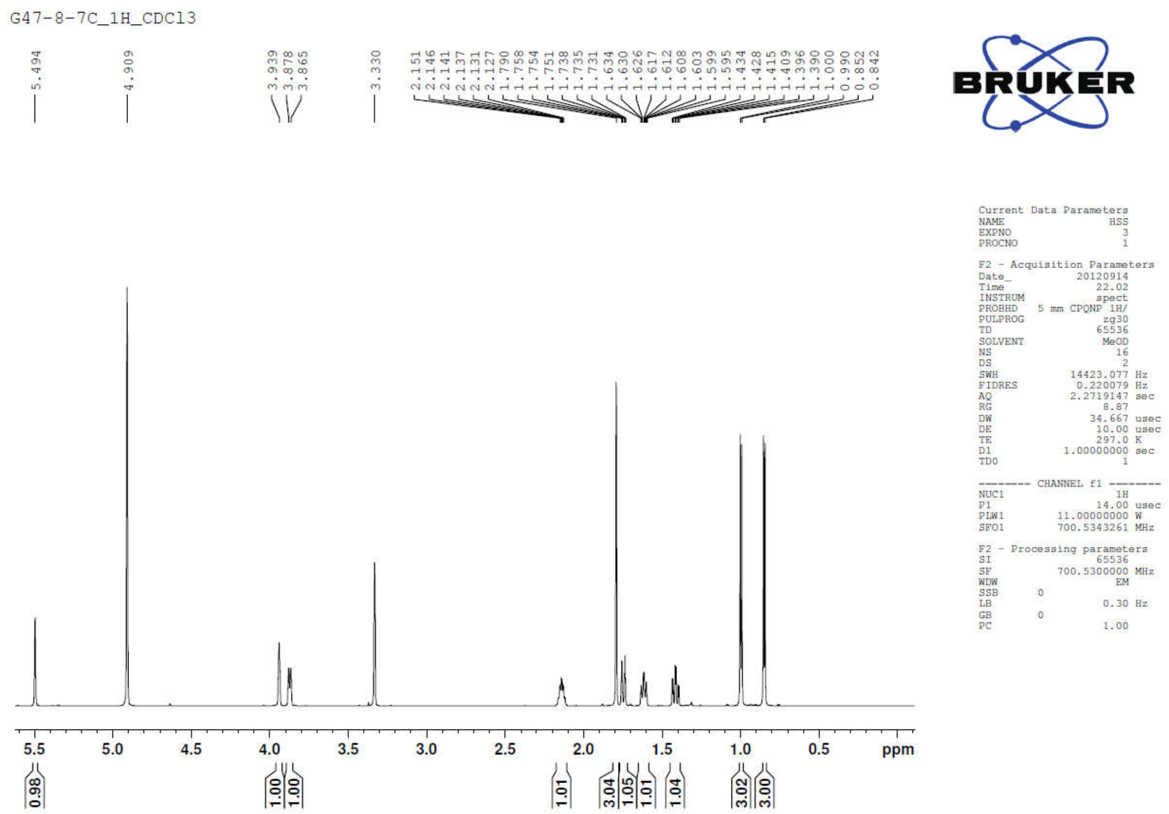

Figure S5.

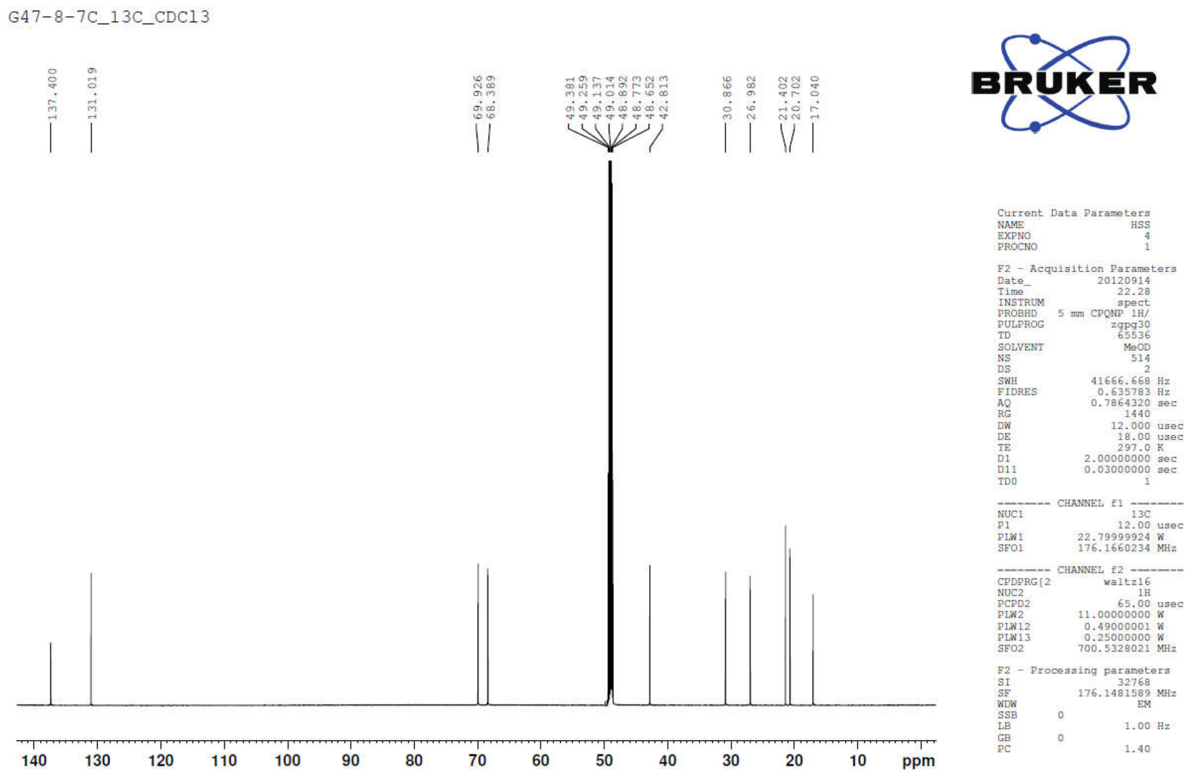

Figure S6.

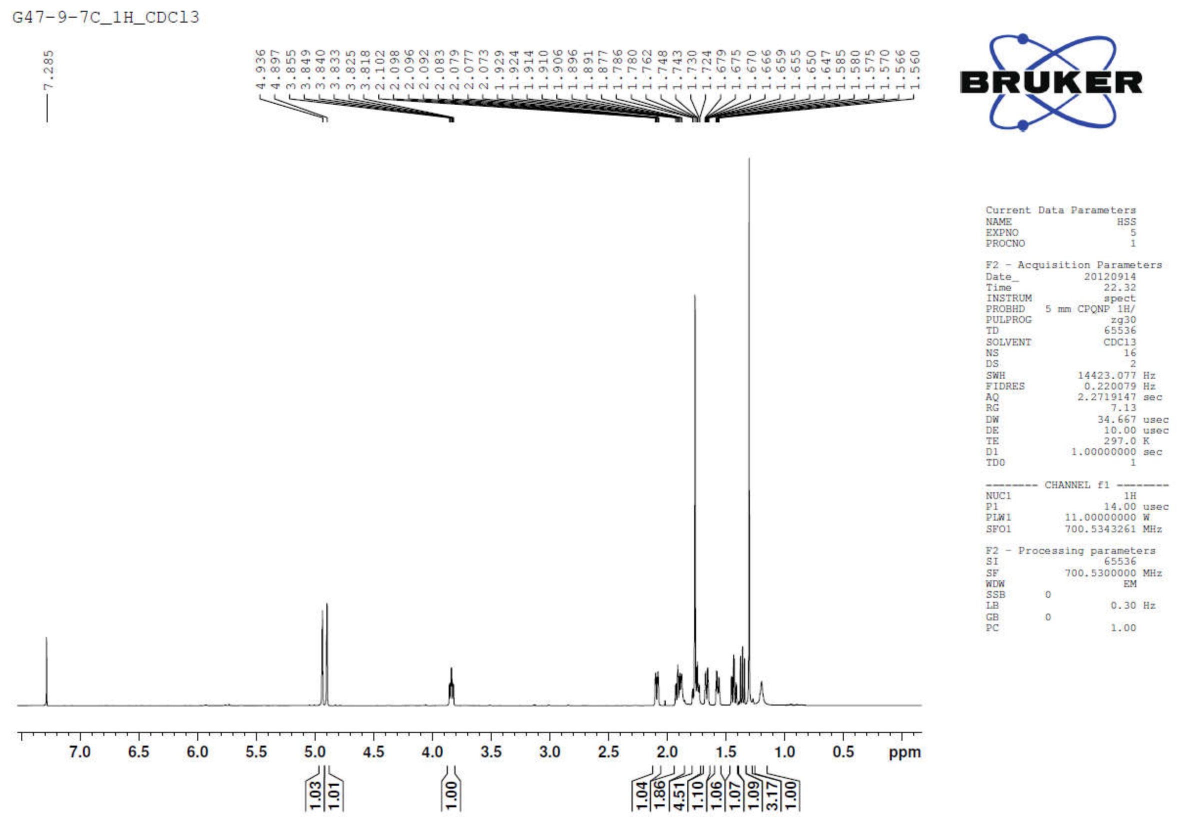

Figure S7.

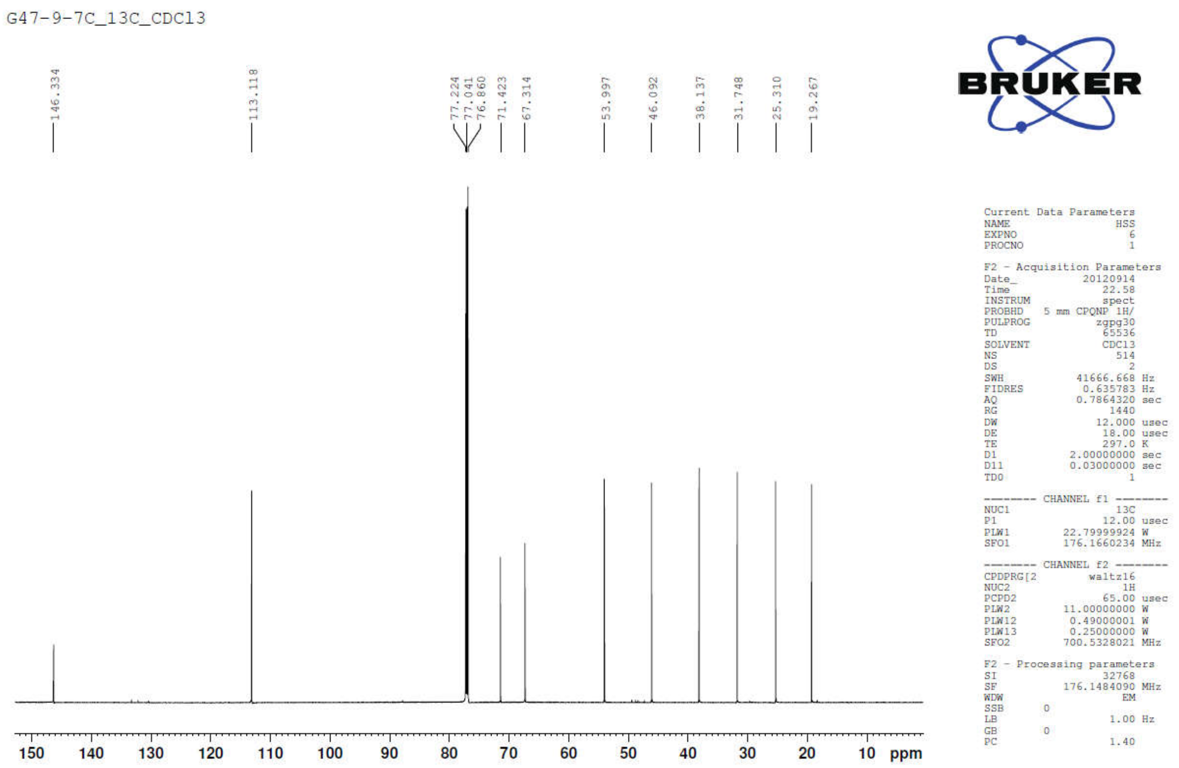

Figure S8.

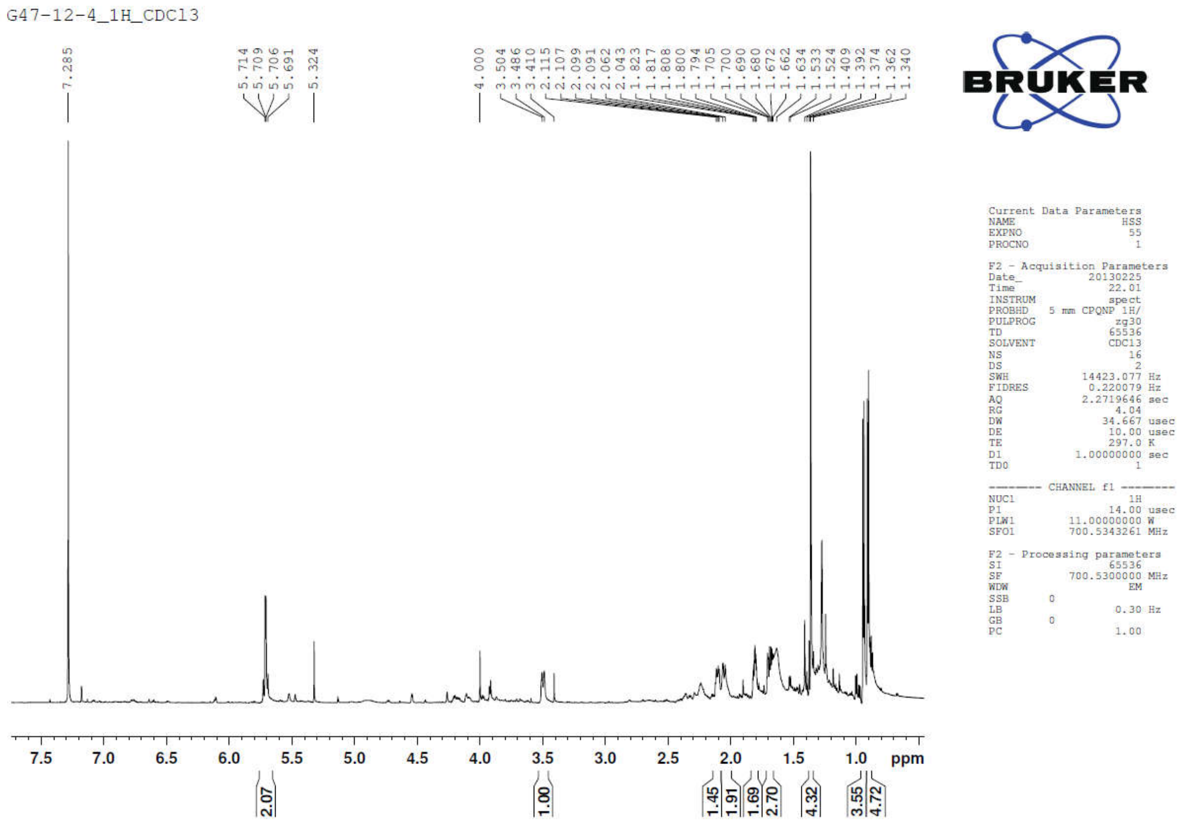

Figure S9.

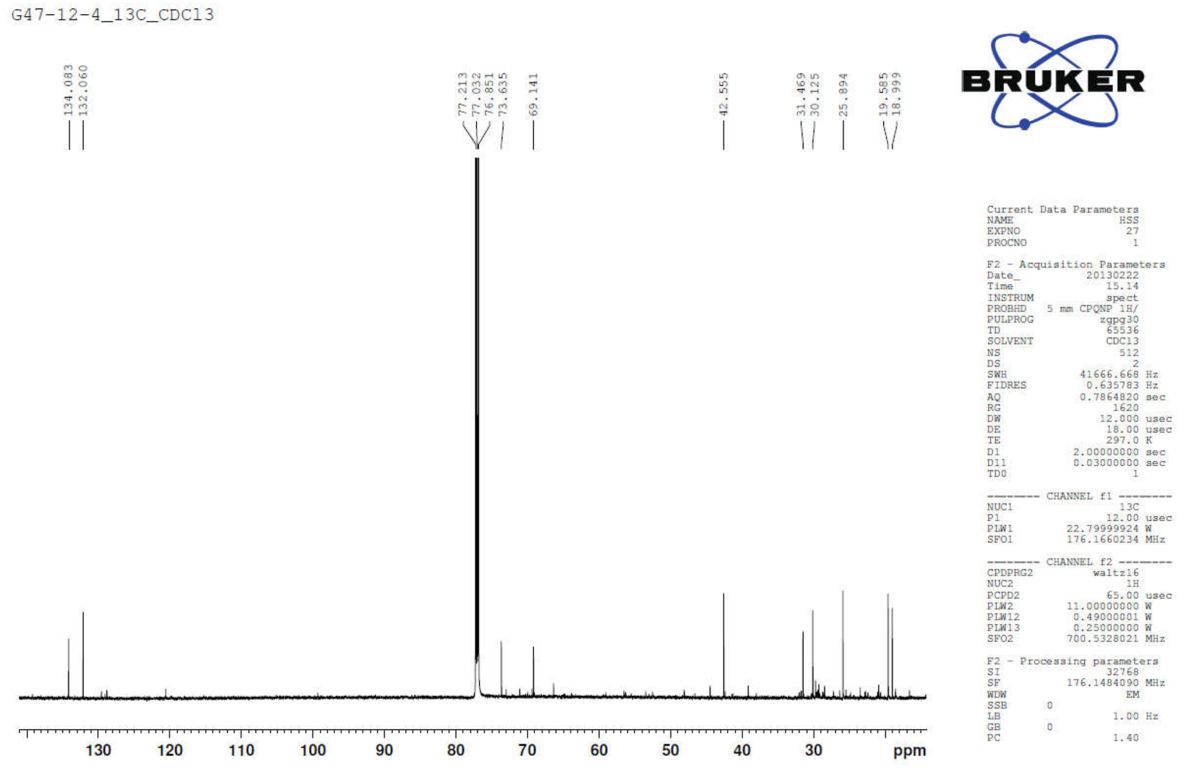

Figure S10.

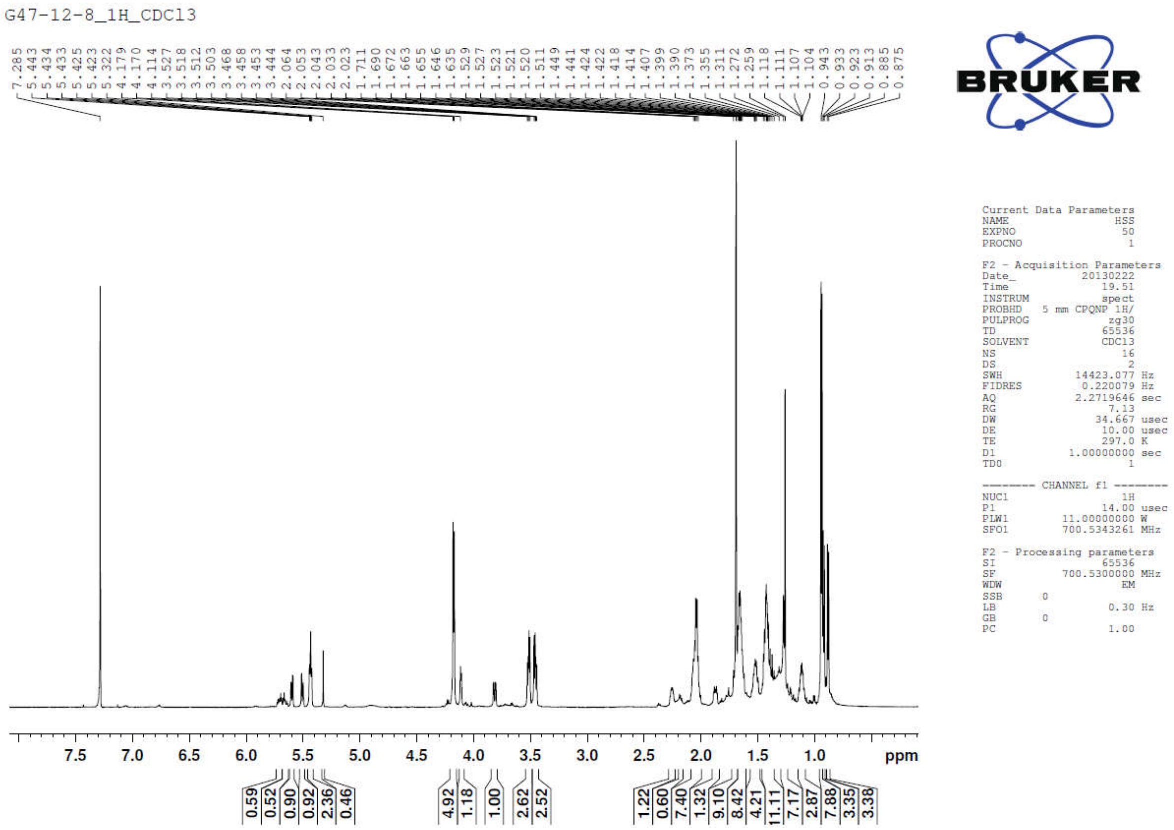

Figure S11.

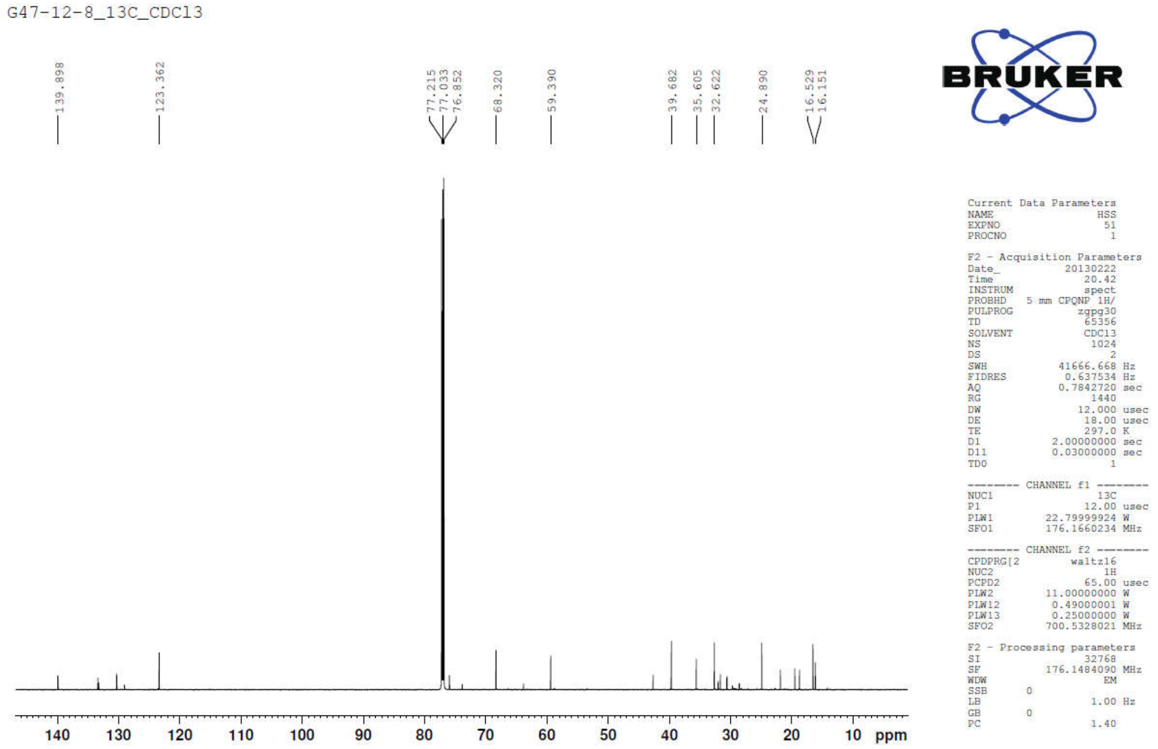

Figure S12.

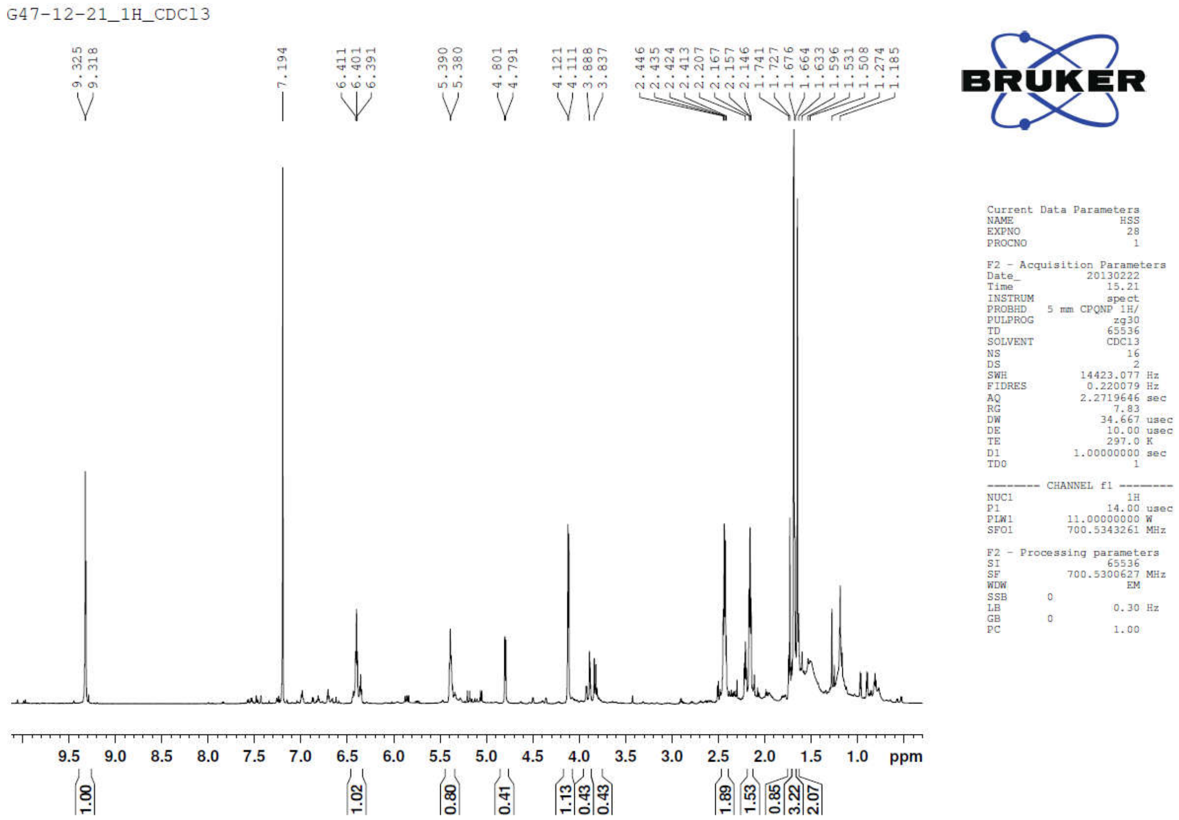

Figure S13.

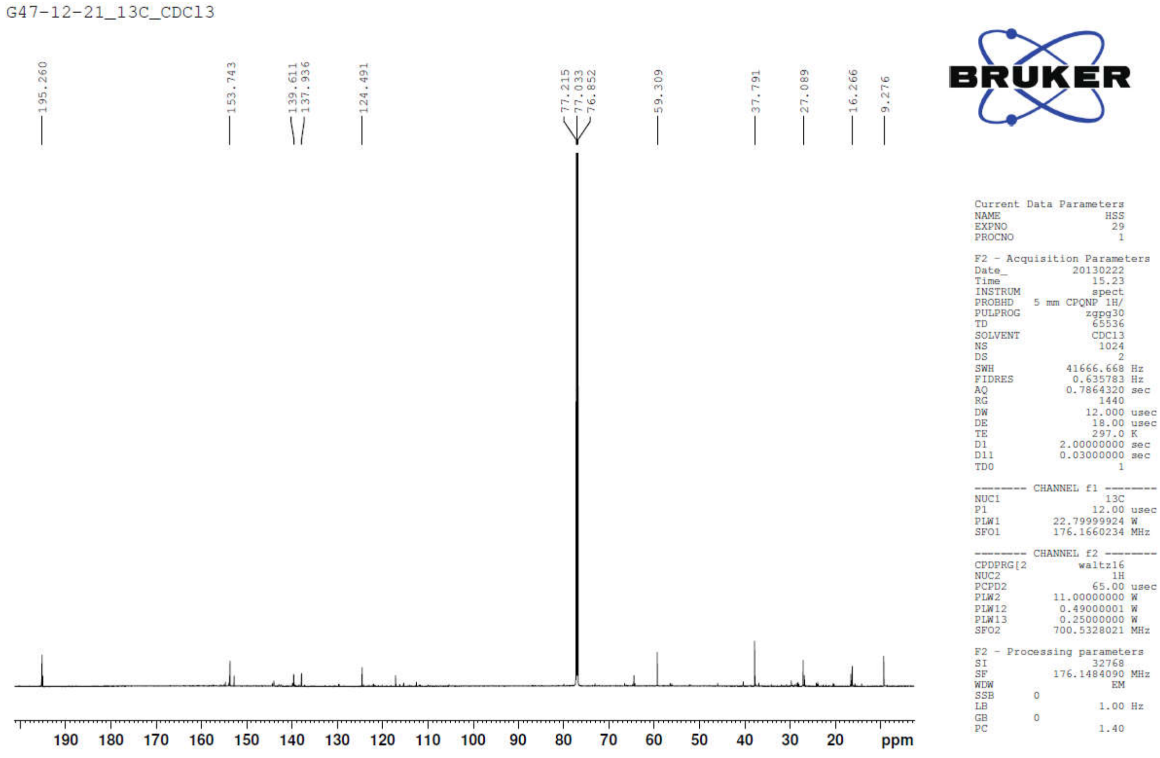

Supplement: Supplementary file 1 [file plants-10-00257-s001.pdf]
